# Cigarette smoking and PM_2.5_ might jointly exacerbate the risk of metabolic syndrome

**DOI:** 10.3389/fpubh.2023.1234799

**Published:** 2024-01-15

**Authors:** Hao-Hung Tsai, Disline Manli Tantoh, Wen Yu Lu, Chih-Yi Chen, Yung-Po Liaw

**Affiliations:** ^1^Institute of Medicine, Chung Shan Medical University, Taichung City, Taiwan; ^2^College of Medicine, Chung Shan Medical University, Taichung City, Taiwan; ^3^Department of Medical Imaging, Chung Shan Medical University Hospital, Taichung City, Taiwan; ^4^Department of Medical Imaging, School of Medicine, Chung Shan Medical University, Taichung City, Taiwan; ^5^Department of Medical Imaging and Radiological Sciences, Chung Shan Medical University, Taichung City, Taiwan; ^6^Department of Public Health and Institute of Public Health, Chung Shan Medical University, Taichung City, Taiwan; ^7^Division of Thoracic Surgery, Department of Surgery, Chung Shan Medical University Hospital, Taichung City, Taiwan

**Keywords:** cigarette smoking, PM_2.5_, interaction, adults, Taiwan

## Abstract

**Background:**

Cigarette smoking and particulate matter (PM) with aerodynamic diameter < 2.5 μm (PM_2.5_) are major preventable cardiovascular mortality and morbidity promoters. Their joint role in metabolic syndrome (MS) pathogenesis is unknown. We determined the risk of MS based on PM_2.5_ and cigarette smoking in Taiwanese adults.

**Methods:**

The study included 126,366 Taiwanese between 30 and 70 years old with no personal history of cancer. The Taiwan Biobank (TWB) contained information on MS, cigarette smoking, and covariates, while the Environmental Protection Administration (EPA), Taiwan, contained the PM_2.5_ information. Individuals were categorized as current, former, and nonsmokers. PM_2.5_ levels were categorized into quartiles: PM_2.5_ ≤ Q1, Q1 < PM_2.5_ ≤ Q2, Q2 < PM_2.5_ ≤ Q3, and PM_2.5_ > Q3, corresponding to PM_2.5_ ≤ 27.137, 27.137 < PM_2.5_ ≤ 32.589, 32.589 < PM_2.5_ ≤ 38.205, and PM_2.5_ > 38.205 μg/m^3^.

**Results:**

The prevalence of MS was significantly different according to PM_2.5_ exposure (*p*-value = 0.0280) and cigarette smoking (*p*-value < 0.0001). Higher PM_2.5_ levels were significantly associated with a higher risk of MS: odds ratio (OR); 95% confidence interval (CI) = 1.058; 1.014–1.104, 1.185; 1.134–1.238, and 1.149; 1.101–1.200 for 27.137 < PM_2.5_ ≤ 32.589, 32.589 < PM_2.5_ ≤ 38.205, and PM_2.5_ > 38.205 μg/m^3^, respectively. The risk of MS was significantly higher among former and current smokers with OR; 95% CI = 1.062; 1.008–1.118 and 1.531; 1.450–1.616, respectively, and a dose-dependent *p*-value < 0.0001. The interaction between both exposures regarding MS was significant (*p*-value = 0.0157). Stratification by cigarette smoking revealed a significant risk of MS due to PM_2.5_ exposure among nonsmokers: OR (95% CI) = 1.074 (1.022–1.128), 1.226 (1.166–1.290), and 1.187 (1.129–1.247) for 27.137 < PM_2.5_ ≤ 32.589, 32.589 < PM_2.5_ ≤ 38.205, and PM_2.5_ > 38.205 μg/m^3^, respectively. According to PM_2.5_ quartiles, current smokers had a higher risk of MS, regardless of PM_2.5_ levels (OR); 95% CI = 1.605; 1.444–1.785, 1.561; 1.409–1.728, 1.359; 1.211–1.524, and 1.585; 1.418–1.772 for PM_2.5_ ≤ 27.137, 27.137 < PM_2.5_ ≤ 32.589, 32.589 < PM_2.5_ ≤ 38.205, and PM_2.5_ > 38.205 μg/m^3^, respectively. After combining both exposures, the group, current smokers; PM_2.5_ > 38.205 μg/m^3^ had the highest odds (1.801; 95% CI =1.625–1.995).

**Conclusion:**

PM_2.5_ and cigarette smoking were independently and jointly associated with a higher risk of MS. Stratified analyses revealed that cigarette smoking might have a much higher effect on MS than PM_2.5_. Nonetheless, exposure to both PM_2.5_ and cigarette smoking could compound the risk of MS.

## Background

Metabolic syndrome (MS) is a condition characterized by the coexistence of at least three metabolic risk markers, including impaired fasting blood glucose (sugar), dyslipidemia (low high-density cholesterol and high triglyceride), abdominal obesity (high waist circumference), and elevated blood pressure (1–4). MS is a public health challenge with a huge global burden: it enhances morbidity and mortality related to chronic diseases such as cancer, stroke, diabetes, asthma, and atherosclerotic and nonatherosclerotic cardiovascular disease (5–7). Metabolic risk factors such as blood pressure, fasting plasma glucose, and high total cholesterol were among the ten largest contributors to global disability-adjusted life years (DALYs) in 2015 (8). MS has multiple promoting factors including, age (9), unhealthy diet (10, 11), obesity (11), alcohol consumption (12, 13), physical inactivity (11, 13), cigarette smoking (13–23), and PM_2.5_ (24–27).

Cigarette smoking is a major preventable promoter of global cardiovascular mortality and morbidity (16, 28, 29). In 2015, it was among the five top risk factors attributable to global DALYs in 109 countries (8). The influence of cigarette smoking on MS and its components is contentious (14, 30). For instance, cigarette smoking was a significant cause of MS among Chinese (14), Koreans (31–33), and Japanese (18, 23). Nonetheless, it was not significantly associated with MS among Japanese (34) and Chinese (35). Furthermore, heavy cigarette smoking among Turkish women was suggested as being protective against future MS (36).

Air pollution, especially PM_2.5_ (fine PM) is an urgent global public health concern, with continuously increasing implications (4, 9, 37–46). It significantly enhances neurological and cardiovascular morbidity and mortality (47, 48). Several studies reported contrasting findings regarding the relationship between MS and PM_2.5_ (14–23, 31–33, 49–51). A recent systematic review and meta-analysis found that PM_2.5_ could contribute to as much as 12.28% of MS (52). In several original studies, PM_2.5_ exposure significantly elevated the risk of MS among Chinese (25–27, 53, 54), Saudi (55), and Korean adults (56). On the contrary, PM_2.5_ did not significantly affect the risk of MS among Germans (57) and Chinese (50).

The positive association between PM_2.5_ and MS was more prominent in cigarette smokers, alcohol drinkers, and obese people (25, 26, 53). This suggests that smoking and other unhealthy habits could exacerbate the adverse effects of air pollution (25, 26, 53). Smoking could also confound the effect of air pollution on cardiovascular health (58). Hence, pinpointing the combined effect of cigarette smoking and PM_2.5_ could narrow the data gap for the burden of disease attributable to both exposures (59). Moreover, determining the interaction between PM_2.5_ and smoking could provide insightful knowledge regarding the susceptibility to PM_2.5_-related adverse health conditions in smokers and nonsmokers (59). High exposure to PM_2.5_ among Chinese was recently associated with a higher risk of hypertension caused by smoking (60). However, robust studies have not been conducted to determine the combined effect of PM_2.5_ and cigarette smoking on MS. In the current study, we determined the independent association of ambient PM_2.5_ and smoking with MS in Taiwanese adults. Moreover, we determined the interaction between PM_2.5_ and smoking regarding MS.

## Methods

### Study participants and data acquisition

We acquired information relating to MS, cigarette smoking, sex, age, weight, height, alcohol drinking, exercise, marital status, educational level, secondhand smoke exposure, and duration of residence from the TWB (2008–2020). The TWB database is one of the human biological databases currently supplying data for biomedical research in Taiwan (61). The TWB project is a community-based prospective study whose participants are exclusively Taiwanese adults with no personal history of cancer (62, 63). At the start of the project, only Taiwanese aged 30–70 were eligible for enrolment (63). Currently, individuals between 20 and 70 years old without a diagnosis of cancer can enroll in the project (62). The TWB biobank currently contains over 30 recruitment sites all over Taiwan (62). All volunteers sign informed consent forms before enrolment. At enrolment, each volunteer fills out the TWB questionnaire, undergoes anthropometric examinations, and provides blood/urine samples for biochemical testing. The questionnaire contains information on cigarette smoking, sex, age, alcohol drinking, exercise, etc. The anthropometry examination determines weight, height, waist circumference, and blood pressure. The biochemical tests determine fasting blood glucose (FBG), triglyceride (TG), and high-density lipoprotein cholesterol (HDL-C), among others.

Currently, the TWB database lacks PM_2.5_ data. Notwithstanding, the Taiwan Environmental Protection Administration (EPA) contains about 71 automated stations that record daily average PM_2.5_ concentrations. We used the EPA daily average data from 2000 to 2016 and computed the annual average PM_2.5_ concentrations (μg/m^3^). The spatial–temporal variability of PM_2.5_ in 349 areas in Taiwan was assessed using machine learning-coupled land-use regression (LUR) as previously described (64). The PM_2.5_ data for each area was considered the exposure data for the participants’ current residing there. The initial study sample was 131,498. However, we excluded 5,132 individuals with missing information for at least one variable. The final analysis included 126,366 people with complete data. The Institutional Review Board (IRB) of the Chung Shan Medical University Hospital granted ethical approval for this study (IRB: CS1-20009).

### Definition of variables

MS was defined as the presence of at least three of the following metabolic markers: (1) waist circumference ≥ 90 cm in men or ≥ 80 cm in women; (2) systolic blood pressure (SBP) ≥ 130 mmHg or diastolic blood pressure (DBP) ≥ 85 mmHg; (3) FBG ≥ 100 mg/dL; (4) HDL-C < 40 mg/dL for men and < 50 mg/dL for women; (5) triglyceride (TG) ≥ 150 mg/dL. This definition was based on the guidelines of the Health Promotion Administration, Ministry of Health and Welfare, Taiwan. Mean annual PM_2.5_ concentrations between 2000 and 2016 were grouped into quartiles: PM_2.5_ ≤ Q1 (PM_2.5_ ≤ 27.137 μg/m^3^), Q1 < PM_2.5_ ≤ Q2 (27.137 < PM_2.5_ ≤ 32.589 μg/m^3^), Q2 < PM_2.5_ ≤ Q3 (32.589 < PM_2.5_ ≤ 38.205 μg/m^3^), and PM_2.5_ > Q3 (PM_2.5_ > 38.205 μg/m^3^). Smoking habits were self-reported, and individuals were categorized as current, former, or nonsmokers. Current smokers included those who smoked cigarettes for at least six months and were still smoking during the data collection period. Former smokers were those who smoked cigarettes for at least six months in the past but had quit the habit for over six months. Nonsmokers were those with no personal history of cigarette smoking.

The body mass index (BMI) was computed as weight/height squared (kg/m^2^). The cutoff values for BMI categories were BMI < 18.5, 18.5 ≤ BMI < 24, 24 ≤ BMI < 27, and BMI ≥ 27 kg/m^2^, corresponding to normal weight, underweight, overweight, and obesity. Current drinkers were individuals who confirmed having a regular habit of consuming at least 150 mL of alcohol per week continuously for half a year or more. Former drinkers included those who drank 150 mL of alcohol per week continuously for at least half a year in the past but had quit the habit for over six months. Nondrinkers included those who drank <150 mL of alcohol per week. Physically active individuals included those who had a habit of regularly engaging in physical activities (lasting over half an hour) at least three times weekly. Exposure to secondhand smoke referred to habitual exposure to secondhand smoke for 5 min or more in an hour. For marital status, participants were regarded as being married (still married), single, divorced/separated (not yet married/divorced or separated from their spouses), or widowed (lost a partner). Educational level categories included, elementary and below, junior and senior high school, or university and above. The quartiles for the duration of residence were < 7.58, 7.58–17.58, 17.58–29.58, and ≥ 29.58 years.

### Statistical analyses

The differences in age (a continuous variable) between participants with and without MS were determined with the Student t-test. The differences in the percentage distribution of categorical variables (e.g., sex, cigarette smoking) between those with and without MS were determined using the Chi-square test. Age was presented in mean ± standard error (SE) while the categorical variables were presented as *n* (%). The risk of MS based on PM_2.5_, cigarette smoking, and the interaction between both exposures was determined by multivariate logistic regression. In the logistic regression model assessing the interaction between cigarette smoking and PM_2.5_ on MS, the *p*-value was obtained by putting the interaction term (cigarette smoking*PM_2.5_) as the main exposure (independent variable) and MS as the outcome variable. In all the regression analyses, adjustments were made for sex, age, weight, height, alcohol drinking, exercise, marital status, educational level, secondhand smoke exposure, and duration of residence. A *p*-value < 0.05 was set as the threshold for statistical significance. Data were managed and analyzed using SAS 9.4 (SAS Institute Inc., Cary, NC).

## Results

Table 1 shows the demographic characteristics of the 126,366 study participants comprising 26,767 MS cases and 99,599 individuals without MS. The PM_2.5_ quartiles were PM_2.5_ ≤ Q1 (PM_2.5_ ≤ 27.137), Q1 < PM_2.5_ ≤ Q2 (27.137 < PM_2.5_ ≤ 32.589 μg/m^3^), Q2 < PM_2.5_ ≤ Q3 (32.589 < PM_2.5_ ≤ 38.205 μg/m^3^), and PM_2.5_ > Q3 (PM_2.5_ > 38.205 μg/m^3^). Individuals with and without MS significantly differed in terms of PM_2.5_ concentration (*p*-value = 0.0280), cigarette smoking, and other variables, including sex, age, BMI, alcohol intake, marital status, educational level, secondhand smoke exposure, and duration of residence (*p*-value < 0.0001). Among the 99,599 individuals without MS, 24,378 (24.48%), 27,157 (27.27%), 23,147 (23.24%), 24,917 (25.02%) were within the PM_2.5_ quartiles, PM_2.5_ ≤ Q1, Q1 < PM_2.5_ ≤ Q2, Q2 < PM_2.5_ ≤ Q3, and PM_2.5_ > Q3, respectively. Among the 26,767 MS cases, 6,370 (23.80%), 7,250 (27.09%), 6,414 (23.96%), and 6,733 (25.15%) were within the PM_2.5_ quartiles, PM_2.5_ ≤ Q1, Q1 < PM_2.5_ ≤ Q2, Q2 < PM_2.5_ ≤ Q3, and PM_2.5_ > Q3, respectively. The group without MS comprised 81,706 (82.03%) nonsmokers, 9,687 (9.73%) former smokers, and 8,206 (8.24%) current smokers. The MS group comprised 19,541 (73.00%) nonsmokers, 3,628 (13.55%) former smokers, and 3,598 (13.44%) nonsmokers.

**Table 1:** Demographic characteristics of the study participants stratified by metabolic syndrome.

Variables	No metabolic syndrome	Metabolic syndrome	*p*-value
	(*n* = 99,599)	(*n* = 26,767)	
PM_2.5_ quartile, *n* (%)			0.0280
PM_2.5_ ≤ Q1 (PM_2.5_ ≤ 27.137 μg/m^3^)	24,378 (24.48)	63,70 (23.80)	
Q1 < PM_2.5_ ≤ Q2 (27.137 < PM_2.5_ ≤ 32.589 μg/m^3^)	27,157 (27.27)	7,250 (27.09)	
Q2 < PM_2.5_ ≤ Q3 (32.589 < PM_2.5_ ≤ 38.205 μg/m^3^)	23,147 (23.24)	64,14 (23.96)	
PM_2.5_ > Q3 (PM_2.5_ > 38.205 μg/m^3^)	24,917 (25.02)	6,733 (25.15)	
Cigarette smoking status, *n* (%)			<0.0001
Nonsmokers	81,706 (82.03)	19,541 (73.00)	
Former smokers	9,687 (9.73)	3,628 (13.55)	
Current smokers	8,206 (8.24)	3,598 (13.44)	
Sex, *n* (%)			<0.0001
Women	6,5,749 (66.01)	14,973 (55.94)	
Men	33,850 (33.99)	11,794 (44.06)	
Age, *n* (%)			<0.0001
Age < 50 years	50,614 (50.82)	8,935 (33.38)	
Age ≥ 50 years	48,985 (49.18)	17,832 (66.62)	
BMI, *n* (%)			<0.0001
Underweight (BMI < 18.5 kg/m^2^)	4,192 (4.21)	26 (0.10)	
Normal weight (18.5 ≤ BMI < 24 kg/m^2^)	57,268 (57.50)	4,682 (17.49)	
Overweight (24 ≤ BMI < 27 kg/m^2^)	25,174 (25.28)	9,037 (33.76)	
Obesity (BMI ≥ 27 kg/m^2^)	12,965 (13.02)	13,022 (48.65)	
Alcohol intake status, *n* (%)			<0.0001
Nondrinkers	9,1946 (93.32)	23,573 (88.07)	
Former drinkers	2,323 (2.33)	1,012 (3.78)	
Current drinkers	5,330 (5.35)	2,182 (8.15)	
Exercise, *n* (%)			0.1665
No	59,543 (59.78)	16,127 (60.25)	
Yes	40,056 (40.22)	10,640 (39.75)	
Marital status, *n* (%)			<0.0001
Married	72,256 (72.55)	19,849 (74.15)	
Single	15,113 (15.17)	2,804 (10.48)	
Divorced or separated	8,251 (8.28)	2,501 (9.34)	
Widowed	3,979 (4.00)	1,613 (6.03)	
Educational level, *n* (%)			<0.0001
Elementary school and below	3,924 (3.94)	2,189 (8.18)	
Junior and senior high school	34,053 (34.19)	11,330 (42.33)	
University and above	61,622 (61.87)	13,248 (49.49)	
Secondhand smoke exposure, *n* (%)			<0.0001
No	89,566 (89.93)	23,529 (87.90)	
Yes	10,033 (10.07)	3,238 (12.10)	
Duration of residence, *n* (%)			<0.0001
<7.58 years	26,129 (26.23)	5,448 (20.35)	
7.58–17.58 years	25,695 (25.80)	5,847 (21.84)	
17.58–29.58 years	24,310 (24.41)	7,143 (26.69)	
≥29.58 years	23,465 (23.56)	8,329 (31.12)	

*n*, sample size; %, percent; BMI, body mass index; kg, kilogram; m^2^, meter squared.

Table 2 and Supplementary Figures S1, S2 present the association of MS with PM_2.5_ and cigarette smoking. Higher compared to lower PM_2.5_ levels (27.137 < PM_2.5_ ≤ 32.589, 32.589 < PM_2.5_ ≤ 38.205, and PM_2.5_ > 38.205 vs. PM_2.5_ ≤ 27.137 μg/m^3^) were significantly associated with a higher risk of MS (OR; 95% CI = 1.058; 1.014–1.104 for 27.137 < PM_2.5_ ≤ 32.589 μg/m^3^, 1.185; 1.134–1.238 for 32.589 < PM_2.5_ ≤ 38.205 μg/m^3^, and 1.149; 1.101–1.200 for PM_2.5_ > 38.205 μg/m^3^). Compared to nonsmokers, former and current smokers had a higher risk of MS (OR = 1.062, 95% CI = 1.008–1.118 for former smokers and 1.531; 1.450–1.616 for current smokers). The dose–response relationship between smoking and MS was significant (p-trend <0.0001). According to the quantity of cigarettes smoked, a weekly consumption of ≥140 cigarettes per week was significantly associated with a higher risk of MS in both former and current smokers (Supplementary Table S1). The interaction between PM_2.5_ and cigarette smoking was significant: *p*-value = 0.0157 (Table 2). The risk of MS was also significantly higher among people who were ≥ 50 years (OR = 2.277, 95% CI = 2.190–2.367), overweight (OR; 95% CI = 4.219; 4.056–4.388), obese (OR; 95% CI = 13.232; 12.707–13.778), current alcohol drinkers (OR = 1.162, 95% CI = 1.092–1.236), divorced/separated (OR; 95% CI = 1.097; 1.039–1.159), and widowed (OR; 95% CI = 1.178;1.098–1.264). However, the risk was lower among underweight individuals (OR = 0.084, 95% CI = 0.057–0.124), those who exercised regularly (OR = 0.866, 95% CI = 0.839–0.895), single people (OR; 95% CI = 0.928; 0.882–0.976), those who attained a junior and senior high school level (OR; 95% CI = 0.821; 0.769–0.876), and university education and above (OR = 0.692, 95% CI = 0.648–0.740).

**Table 2:** Association of PM_2.5_ and cigarette smoking with metabolic syndrome.

Variables	OR	95% CI	*p*-value
PM_2.5_ quartile			
PM_2.5_ ≤ 27.137 (ref.)	1		
27.137 < PM_2.5_ ≤ 32.589	1.058	1.014–1.104	0.0096
32.589 < PM_2.5_ ≤ 38.205	1.185	1.134–1.238	<0.0001
PM_2.5_ > 38.205	1.149	1.101–1.200	<0.0001
P-trend	NA		
Cigarette smoking status			
Nonsmokers (ref.)	1		
Former smokers	1.062	1.008–1.118	0.0232
Current smokers	1.531	1.450–1.616	<0.0001
P-trend	<0.0001		
Sex			
Women (ref.)	1		
Men	0.966	0.930–1.003	0.0705
Age			
Age < 50 (ref.)	1		
Age ≥ 50	2.277	2.190–2.367	<0.0001
BMI			
Normal weight (ref.)	1		
Underweight	0.084	0.057–0.124	<0.0001
Overweight	4.219	4.056–4.388	<0.0001
Obesity	13.232	12.707–13.778	<0.0001
Alcohol intake status			
Nondrinkers (ref.)	1		
Former drinkers	1.056	0.968–1.152	0.2198
Current drinkers	1.162	1.092–1.236	<0.0001
Exercise			
No (ref.)	1		
Yes	0.866	0.839–0.895	<0.0001
Marital status			
Married (ref.)	1		
Single	0.928	0.882–0.976	0.0036
Divorced or separated	1.097	1.039–1.159	0.0009
Widowed	1.178	1.098–1.264	<0.0001
Educational level			
Elementary school and below (ref.)	1		
Junior and senior high school	0.821	0.769–0.876	<0.0001
University and above	0.692	0.648–0.740	<0.0001
Secondhand smoke exposure			
No (ref.)	1		
Yes	1.048	0.998–1.100	0.0614
Duration of residence			
<7.58 (ref.)	1		
7.58–17.58	1.065	1.017–1.114	0.0070
17.58–29.58	1.128	1.078–1.182	<0.0001
≥29.58	1.146	1.092–1.203	<0.0001
PM_2.5_*cigarette smoking	*p*-value = 0.0157		

OR, odds ratio; CI, confidence interval; ref., reference; BMI, body mass index; NA, not applicable (the trend is nonlinear).

Table 3 shows the association between PM_2.5_ and MS in current, former, and nonsmokers. PM_2.5_ was significantly associated with a higher risk of MS among nonsmokers: OR = 1.074, 95% CI = 1.022–1.128, 1.226; 1.166–1.290, and 1.187; 1.129–1.247 for 27.137 < PM_2.5_ ≤ 32.589, 32.589 < PM_2.5_ ≤ 38.205, and PM_2.5_ > 38.205 μg/m^3^, respectively.

**Table 3:** Association between PM_2.5_ and metabolic syndrome in current, former, nonsmokers.

Variables	Nonsmokers			Former smokers			Current smokers		
	OR	95% CI	*p*-value	OR	95% CI	*p*-value	OR	95% CI	*p*-value
PM_2.5_ quartile									
PM_2.5_ ≤ 27.137 (ref.)	1								
27.137 < PM_2.5_ ≤ 32.589	1.074	1.022–1.128	0.0047	1.042	0.927–1.172	0.4856	0.983	0.871–1.110	0.7860
32.589 < PM_2.5_ ≤ 38.205	1.226	1.166–1.290	<0.0001	1.075	0.951–1.215	0.2485	1.044	0.917–1.188	0.5187
PM_2.5_ > 38.205	1.187	1.129–1.247	<0.0001	0.975	0.862–1.103	0.6864	1.094	0.962–1.244	0.1714
P-trend	NA			NA			0.1193		
Sex									
Women (ref.)	1								
Men	0.941	0.903–0.981	0.0042	1.179	1.023–1.359	0.0227	1.341	1.171–1.534	<0.0001
Age									
Age < 50 (ref.)	1								
Age ≥ 50	2.403	2.296–2.514	<0.0001	1.913	1.716–2.133	<0.0001	1.915	1.718–2.135	<0.0001
BMI									
Normal weight (ref.)	1								
Underweight	0.074	0.048–0.115	<0.0001	<0.001	<0.001- > 999.999	0.9431	0.226	0.100–0.511	0.0004
Overweight	4.193	4.013–4.381	<0.0001	4.458	3.916–5.074	<0.0001	4.523	3.971–5.153	<0.0001
Obesity	12.508	11.950–13.091	<0.0001	15.036	13.202–17.125	<0.0001	17.416	15.290–19.839	<0.0001
Alcohol intake status									
Nondrinkers (ref.)	1								
Former drinkers	1.181	1.012–1.377	0.0344	1.021	0.888–1.174	0.7728	1.070	0.905–1.265	0.4313
Current drinkers	1.007	0.908–1.117	0.8941	1.287	1.144–1.447	<0.0001	1.238	1.111–1.380	0.0001
Exercise									
No (ref.)	1								
Yes	0.866	0.834–0.898	<0.0001	0.842	0.770–0.920	0.0002	0.853	0.769–0.947	0.0027
Marital status									
Married (ref.)	1								
Single	0.947	0.894–1.003	0.0646	0.989	0.830–1.179	0.9032	0.863	0.755–0.986	0.0308
Divorced or separated	1.059	0.992–1.130	0.0853	1.321	1.128–1.547	0.0006	1.213	1.056–1.394	0.0063
Widowed	1.161	1.078–1.249	<0.0001	1.192	0.879–1.616	0.2574	1.167	0.828–1.646	0.3773
Educational level									
Elementary school and below (ref.)	1								
Junior and senior high school	0.808	0.753–0.868	<0.0001	1.016	0.823–1.254	0.8845	0.776	0.597–1.009	0.0585
University and above	0.678	0.630–0.729	<0.0001	0.891	0.721–1.102	0.2866	0.673	0.516–0.878	0.0035
Secondhand smoke exposure									
No (ref.)	1								
Yes	1.048	0.986–1.115	0.1303	0.962	0.843–1.097	0.5583	1.102	0.993–1.224	0.0677
Duration of residence									
<7.58 (ref.)	1								
7.58–17.58	1.071	1.016–1.130	0.0112	1.044	0.917–1.189	0.5154	1.045	0.927–1.179	0.4729
17.58–29.58	1.115	1.056–1.177	<0.0001	1.105	0.975–1.252	0.1194	1.196	1.054–1.357	0.0057
≥29.58	1.150	1.087–1.216	<0.0001	1.098	0.959–1.258	0.1765	1.092	0.938–1.273	0.2572

OR, odds ratio; CI, confidence interval; ref., reference; BMI, body mass index; NA, not applicable (the trend is nonlinear).

Table 4 illustrates the association between cigarette smoking and MS stratified by PM_2.5_ quartiles. Compared to nonsmokers, the risk of MS was significantly higher in former smokers when the PM_2.5_ concentration was ≤32.589 μg/m^3^: OR (95% CI) = 1.139 (1.027–1.263) for PM_2.5_ ≤ 27.137 μg/m^3^ and 1.138 (1.032–1.254) for 27.137 < PM_2.5_ ≤ 32.589 μg/m^3^. The risk of MS was significantly higher among current smokers, regardless of the PM_2.5_ concentration. The ORs; 95% CIs were 1.605; 1.444–1.785, 1.561; 1.409–1.728, 1.359; 1.211–1.524; and 1.585; 1.418–1.772 for PM_2.5_ ≤ 27.137, 27.137 < PM_2.5_ ≤ 32.589, 32.589 < PM_2.5_ ≤ 38.205, and PM_2.5_ > 38.205 μg/m^3^, respectively.

**Table 4:** Association between cigarette smoking and metabolic syndrome stratified by PM_2.5_ quartiles.

Variables	PM_2.5_ ≤ 27.137 μg/m^3^			27.137 < PM_2.5_ ≤ 32.589 μg/m^3^			32.589 < PM_2.5_ ≤ 38.205 μg/m^3^			PM_2.5_ > 38.205 μg/m^3^		
	OR	95% CI	*p*-value	OR	95% CI	*p*-value	OR	95% CI	*p*-value	OR	95% CI	*p*-value
Cigarette smoking status												
Nonsmokers (ref.)	1											
Former smokers	1.139	1.027–1.263	0.0139	1.138	1.032–1.254	0.0094	0.960	0.863–1.068	0.4513	1.003	0.901–1.116	0.9590
Current smokers	1.605	1.444–1.785	<0.0001	1.561	1.409–1.728	<0.0001	1.359	1.211–1.524	<0.0001	1.585	1.418–1.772	<0.0001
P-trend	<0.0001			<0.0001			<0.0001			<0.0001		
Sex												
Women (ref.)	1											
Men	0.976	0.904–1.054	0.5371	0.935	0.870–1.005	0.0689	1.056	0.978–1.140	0.1624	0.912	0.846–0.984	0.0177
Age												
Age < 50 (ref.)	1											
Age ≥ 50	2.349	2.169–2.543	<0.0001	2.159	2.002–2.329	<0.0001	2.315	2.139–2.505	<0.0001	2.295	2.126–2.477	<0.0001
BMI												
Normal weight (ref.)	1											
Underweight	0.113	0.054–0.239	<0.0001	0.048	0.018–0.128	<0.0001	0.127	0.066–0.246	<0.0001	0.064	0.029–0.143	<0.0001
Overweight	4.381	4.030–4.762	<0.0001	4.272	3.960–4.609	<0.0001	4.228	3.901–4.583	<0.0001	4.060	3.762–4.381	<0.0001
Obesity	14.795	13.600–16.095	<0.0001	13.166	12.182–14.231	<0.0001	13.506	12.424–14.683	<0.0001	11.850	10.944–12.831	<0.0001
Alcohol intake status												
Nondrinkers (ref.)	1											
Former drinkers	1.052	0.874–1.266	0.5931	1.059	0.890–1.260	0.5167	1.112	0.932–1.327	0.2372	1.033	0.876–1.218	0.7031
Current drinkers	1.165	1.028–1.319	0.0163	1.113	0.993–1.247	0.0654	1.222	1.075–1.389	0.0021	1.171	1.025–1.339	0.0204
Exercise												
No (ref.)	1											
Yes	0.834	0.780–0.891	<0.0001	0.883	0.829–0.939	<0.0001	0.853	0.797–0.912	<0.0001	0.892	0.836–0.951	0.0005
Marital status												
Married (ref.)	1											
Single	0.943	0.847–1.049	0.2788	0.880	0.798–0.970	0.0100	0.898	0.809–0.995	0.0405	0.995	0.902–1.098	0.9218
Divorced or separated	1.118	1.002–1.248	0.0460	1.027	0.923–1.142	0.6282	1.205	1.072–1.355	0.0018	1.065	0.958–1.184	0.2417
Widowed	1.152	0.999–1.329	0.0515	1.172	1.026–1.340	0.0196	1.332	1.150–1.543	0.0001	1.088	0.946–1.252	0.2364
Educational level												
Elementary school and below (ref.)	1											
Junior and senior high school	0.808	0.705–0.925	0.0020	0.814	0.719–0.921	0.0011	0.819	0.720–0.930	0.0021	0.836	0.729–0.958	0.0100
University and above	0.692	0.601–0.795	<0.0001	0.683	0.602–0.776	<0.0001	0.722	0.633–0.824	<0.0001	0.674	0.586–0.776	<0.0001
Secondhand smoke exposure												
No (ref.)	1											
Yes	1.088	0.984–1.203	0.0985	1.065	0.971–1.168	0.1787	1.032	0.940–1.133	0.5140	1.002	0.901–1.114	0.9760
Duration of residence												
<7.58 (ref.)	1											
7.58–17.58	1.038	0.945–1.139	0.4379	1.120	1.022–1.226	0.0149	1.149	0.958–1.149	0.2990	1.053	0.963–1.151	0.2576
17.58–29.58	1.079	0.983–1.185	0.1097	1.166	1.064–1.278	0.0010	1.175	1.071–1.290	0.0007	1.096	1.001–1.200	0.0476
≥29.58	1.080	0.980–1.191	0.1198	1.215	1.106–1.335	<0.0001	1.160	1.046–1.286	0.0048	1.129	1.027–1.242	0.0119

OR, odds ratio; CI, confidence interval; ref., reference; BMI, body mass index.

Table 5 and Supplementary Figure S3 show the risk of MS according to cigarette smoking and PM_2.5_ exposure. Compared to nonsmokers with low PM_2.5_ exposure (PM_2.5_ ≤ 27.137 μg/m^3^), the risk of MS was significantly higher in all the categories. Of note, the category comprising current smokers and PM_2.5_ > 38.205 μg/m^3^ had the highest risk of MS (OR = 1.801, 95% CI = 1.625–1.995).

**Table 5:** Risk of metabolic syndrome based on a combination of cigarette smoking and PM_2.5_ exposure.

Variables	OR	95% CI	*p*-value
Cigarette smoking status and PM_2.5_ exposure			
Nonsmokers; PM_2.5_ ≤ 27.137 (ref.)	1		
Nonsmokers; 27.137 < PM_2.5_ ≤ 32.589	1.075	1.023–1.129	0.0043
Nonsmokers; 32.589 < PM_2.5_ ≤ 38.205	1.224	1.164–1.288	<0.0001
Nonsmokers; PM_2.5_ > 38.205	1.190	1.132–1.250	<0.0001
Former smokers; PM_2.5_ ≤ 27.1374	1.155	1.051–1.270	0.0029
Former smokers; 27.137 < PM_2.5_ ≤ 32.589	1.194	1.091–1.307	0.0001
Former smokers; 32.589 < PM_2.5_ ≤ 38.205	1.248	1.132–1.376	<0.0001
Former smokers; PM_2.5_ > 38.205	1.136	1.029–1.254	0.0114
Current smokers; PM_2.5_ ≤ 27.137	1.648	1.498–1.813	<0.0001
Current smokers; 27.137 < PM_2.5_ ≤ 32.589	1.630	1.484–1.791	<0.0001
Current smokers; 32.589 < PM_2.5_ ≤ 38.205	1.758	1.583–1.953	<0.0001
Current smokers; PM_2.5_ > 38.205	1.801	1.625–1.995	<0.0001
Sex			
Women (ref.)	1		
Men	0.966	0.931–1.004	0.0755
Age			
Age < 50 (ref.)	1		
Age ≥ 50	2.277	2.191–2.367	<0.0001
BMI			
Normal weight (ref.)	1		
Underweight	0.084	0.057–0.124	<0.0001
Overweight	4.219	4.057–4.389	<0.0001
Obesity	13.239	12.714–13.786	<0.0001
Alcohol intake			
Nondrinkers (ref)	1		
Former drinkers	1.060	0.971–1.156	0.1929
Current drinkers	1.163	1.093–1.238	<0.0001
Exercise			
No (ref.)	1		
Yes	0.867	0.839–0.895	<0.0001
Marital status			
Married (ref.)	1		
Single	0.927	0.881–0.975	0.0033
Divorced or separated	1.097	1.038–1.158	0.0010
Widowed	1.178	1.098–1.264	<0.0001
Educational level			
Elementary school and below (ref.)	1		
Junior and senior high school	0.821	0.769–0.876	<0.0001
University and above	0.692	0.647–0.740	<0.0001
Secondhand smoke exposure			
No (ref)	1		
Yes	1.047	0.997–1.099	0.0638
Duration of residence			
<7.58 (ref.)	1		
7.58–17.58	1.064	1.017–1.114	0.0072
17.58–29.58	1.128	1.077–1.182	<0.0001
≥29.58	1.146	1.092–1.203	<0.0001

OR, odds ratio; CI, confidence interval; ref., reference; BMI, body mass index.

## Discussion

Cigarette smoking and PM_2.5_ have significant adverse effects on individual and public health. A systematic analysis of the global burden of disease ranked PM_2.5_ and cigarette smoking among the ten leading causes of death and disability in 2015 (8). We evaluated the independent and joint association of both factors with MS in Taiwan Biobank volunteers. Smoking and PM_2.5_ were independently associated with higher odds of MS. Moreover, both exposures were interactively associated with MS in a significant manner.

Cigarette smoking has been associated with CVD risk factors such as elevated heart rate, dyslipidemia, hyperinsulinemia, and glucose intolerance (15–17). In line with our study, several original studies and meta-analyses reported cigarette smoking as a metabolic syndrome-promoting factor (14–23, 31–33, 51). For instance, in a meta-analysis including 13 prospective studies, active smoking was positively associated with MS (51). In an original study, life-course cigarette smoking was associated with a higher risk of MS among Chinese, particularly those under 70 years (14). Moreover, a cross-sectional study among Koreans below 40 years found a higher likelihood of MS in smokers than nonsmokers (33). Furthermore, a community-based study involving Taiwanese aged 40 years and above revealed a dose-dependent positive relationship of current smoking with MS and some of its components, including high TG and low HDL (22). In addition, a study among Japanese aged 35–65 also showed a higher incidence of MS among both current and former smokers (23). Another study among Japanese between 20 and 93 years found that the risk of MS in individuals who smoked over 40 cigarettes per day persisted even after 20 years of quitting (18). A cross-sectional study among male Korean former smokers aged at least 19 years showed a higher risk of MS, hypertriglyceridemia, and hyperglycemia among those who had smoked for over 20 years (32). Another cross-sectional among male Koreans aged over 20 years also showed a higher risk of MS among former and current smokers who smoked more than ten packs of cigarettes annually (31). In a cross-sectional study involving individuals of Western European ancestry, cigarette smoking was significantly linked to a higher prevalence of MS, regardless of BMI and sex (65). In the DESIR (Données Epidémiologiques sur le Syndrome d’Insulino-Résistance) study (a longitudinal study involving French), male smokers had a significantly higher risk of MS (66). In another longitudinal study in Norway, heavy smoking increased the incidence of MS in both men and women (13). Using the Third National Health and Nutrition Examination Survey (NHANES) data, a study in the US found a lower risk of MS among normal weight and overweight men and women with no history of smoking (67).

The positive association of PM_2.5_ and MS in the current study is comparable to findings from previous studies (14–23, 31–33, 51). For example, exposure to PM_2.5_ exacerbated the risk of MS among Saudi adults (55) and Korean adults without CVDs (56). Moreover, several original studies found a positive relationship between long-term exposure to PM_2.5_ and MS in adult Chinese (25–27, 53, 54). A meta-analysis of observational studies revealed a borderline positive association between PM_2.5_ and MS (49). Exposure to PM_2.5_ has also been associated with an elevated risk of MS components, including high abdominal obesity (56), FBG (54–56, 68–70), high BP (55, 56, 71), and dyslipidemia (54, 56, 70). Analyses of data from the Heinz Nixdorf Recall (HNR) cohort study in Germany revealed a borderline positive association between PM_2.5_ and MS (57). A study in the US using data from the Normative Aging study found a significantly increased risk of MS due to increasing PM_2.5_ concentrations (70). Nonetheless, data from the Adolescent to Adult Health (Add Health) study (a longitudinal study in the US) showed no significant association between long-term PM_2.5_ exposure and MS (72).

In our study, the interaction of PM_2.5_ and cigarette smoking on MS was significant. It is worth noting that the joint role of both exposures in MS pathogenesis has not received considerable attention. However, some studies investigated the joint role of PM and cigarette smoking on cardiovascular and pulmonary morbidity and mortality (59, 73–75). For instance, Turner and colleagues (59) reported an increased risk of cardiovascular mortality (i.e., about 32 extra deaths per 100,000 person-years) due to smoking-PM_2.5_ interaction. Even though a study on cardiovascular mortality found no interaction between PM_2.5_ and smoking, current smokers with higher exposure to PM_2.5_ had a high relative risk for mortality (76). Exposure to both smoking and PM_2.5_ was associated with a relative excess risk of lung cancer mortality (74). Exposure to particulate matter, especially PM_2.5_, was also significantly associated with a higher risk of cardio-cerebrovascular disease among nonsmokers (73).

The potential mechanisms underpinning the role of smoking and PM_2.5_ on MS are unclear. Nonetheless, the available evidence points toward insulin resistance, induced oxidative stress, inflammation, and endothelial dysfunction. That is, smoking is believed to promote MS by inducing insulin resistance, reducing insulin sensitivity, and causing hyperglycemia, high blood pressure, hyperinsulinemia, oxidative stress, endothelial dysfunction, and systemic inflammation (15, 16, 77, 78). Air pollution, especially PM_2.5_, enhances MS susceptibility by disrupting insulin signaling, inducing inflammation and oxidative stress (73, 79–82). Sung Kyun Park and colleagues (83) found that in MS patients, PM could particularly affect CVDs by causing cardiac autonomic dysfunction.

The current study has some limitations. First, we included only Taiwanese adults aged 30 and 70 who were enrolled in the TWB project. The restriction of enrolment to only Taiwanese within a specific age cohort is a possible source of selection bias. As such, our conclusions may not be generalizable to non-Taiwanese and Taiwanese outside the 30–70 age group. Second, we could not ascertain PM_2.5_ exposure at individual levels since data were obtained from fixed monitoring stations. The non-definitive ascertainment of smoking and PM_2.5_ exposures could have resulted in measurement error or information bias and consequently, wrong classification. Nonetheless, we believe that the misclassification could be nondifferential as it involved both cases and controls from a community-based cohort. The nondifferential misclassification could have resulted in the underestimation of MS risk. We recommend that the findings from this study should be replicated in other populations. Moreover, studies in Taiwan should consider including adults outside the 30–70 years age group. Furthermore, to get the actual effect of cigarette smoking on MS, future studies should consider the number of cigarettes smoked and determine the levels of cotinine (a biomarker of tobacco consumption).

## Conclusion

Summarily, PM_2.5_ and smoking were independently and interactively associated with a higher risk of MS. Stratified analyses revealed that cigarette smoking might have a much higher effect on MS than PM_2.5_. After integrating smoking and PM_2.5_ exposure in the same model, the risk of MS was highest among current cigarette smokers exposed to the highest level of PM_2.5_. Quitting smoking could reduce the incidence of MS in individuals exposed to PM_2.5_. As PM_2.5_ could affect nonsmokers, targeting it could also be very beneficial in reducing the risk of MS in these individuals. To curb smoking, PM_2.5_, and their adverse effects, the government could enforce stronger and more sustainable policies such as funding mass media campaigns on the dangers of environmental factors. The government could also provide incentives for smoking cessation treatments.

## Data availability statement

The data analyzed in this study is subject to the following licenses/restrictions: the data that support the findings of this study are available from Taiwan Biobank but restrictions apply to the availability of these data, which were used under license for the current study, and so are not publicly available. Data are, however available from the corresponding author, Y-PL upon reasonable request and with permission of Taiwan Biobank. Requests to access these datasets should be directed to Y-PL, Liawyp@csmu.edu.tw.

## Ethics statement

The studies involving humans were approved by the Institutional Review Board (IRB) of the Chung Shan Medical University Hospital granted ethical approval for this study (IRB: CS1-20009). The studies were conducted in accordance with the local legislation and institutional requirements. The participants provided their written informed consent to participate in this study.

## Author contributions

H-HT, DT, WL, C-YC, and Y-PL did the literature search, conceived, and designed the study. WL and Y-PL analyzed the data. H-HT, DT, and C-YC drafted the manuscript. DT edited the paper. All authors contributed to the article and approved the submitted version.
