# Supplementary material for: Cigarette smoking and PM2.5 might jointly exacerbate the risk of metabolic syndrome
Source: Front Public Health. 2024 Jan 15;11:1234799. doi: 10.3389/fpubh.2023.1234799 (PMC10822970; doi:10.3389/fpubh.2023.1234799)
Supplement: Supplementary file 1 [file Data_Sheet_1.pdf]

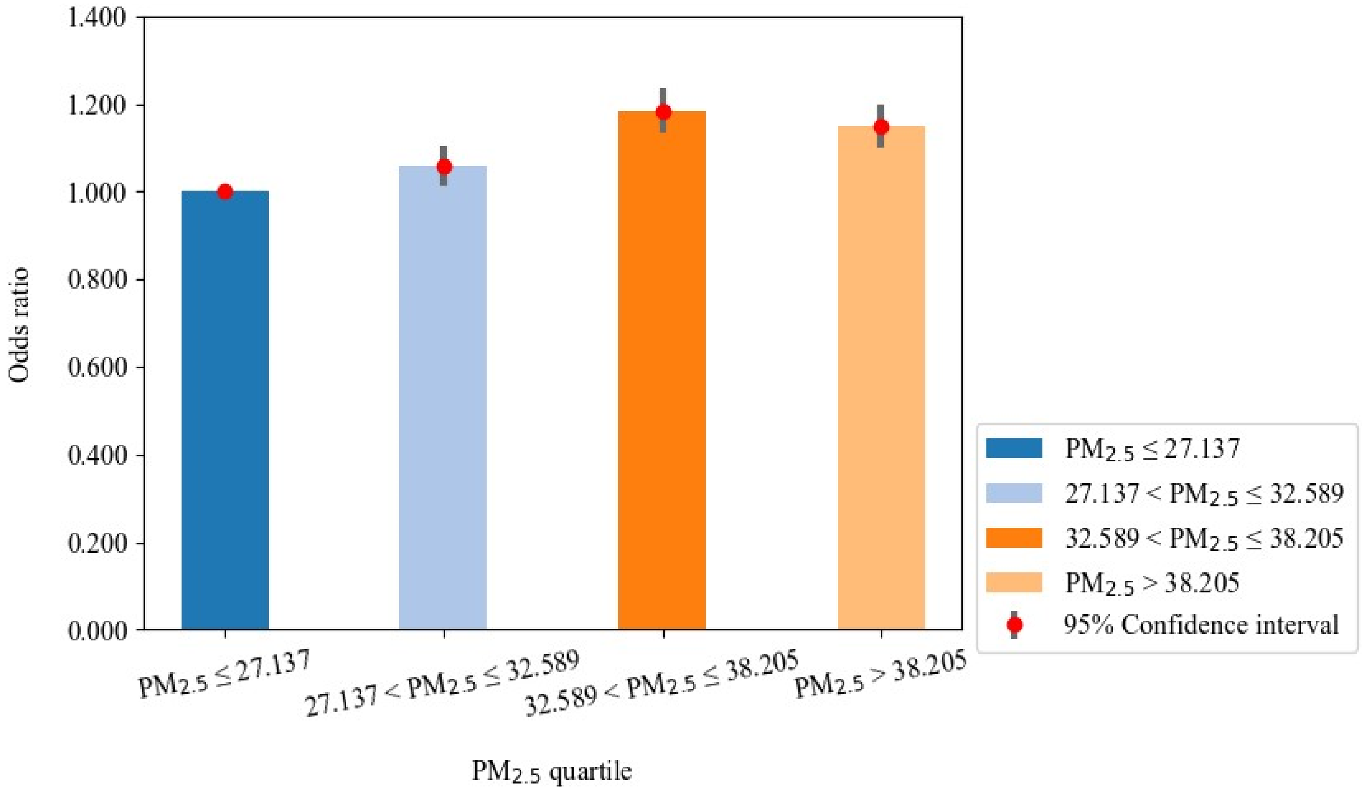

Supplementary figure 1. Association between $PM_{2.5}$ and metabolic syndrome

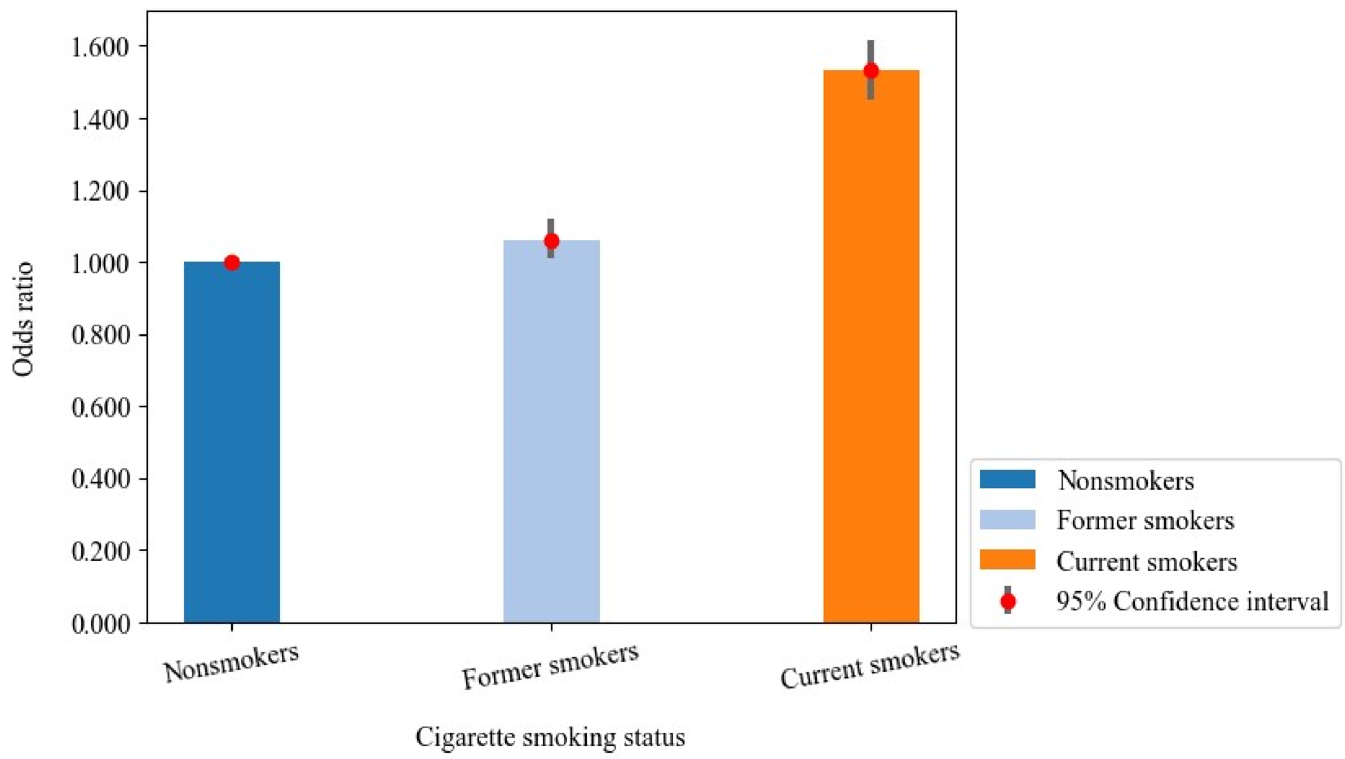

Supplementary figure 2. Association between cigarette smoking and metabolic syndrome

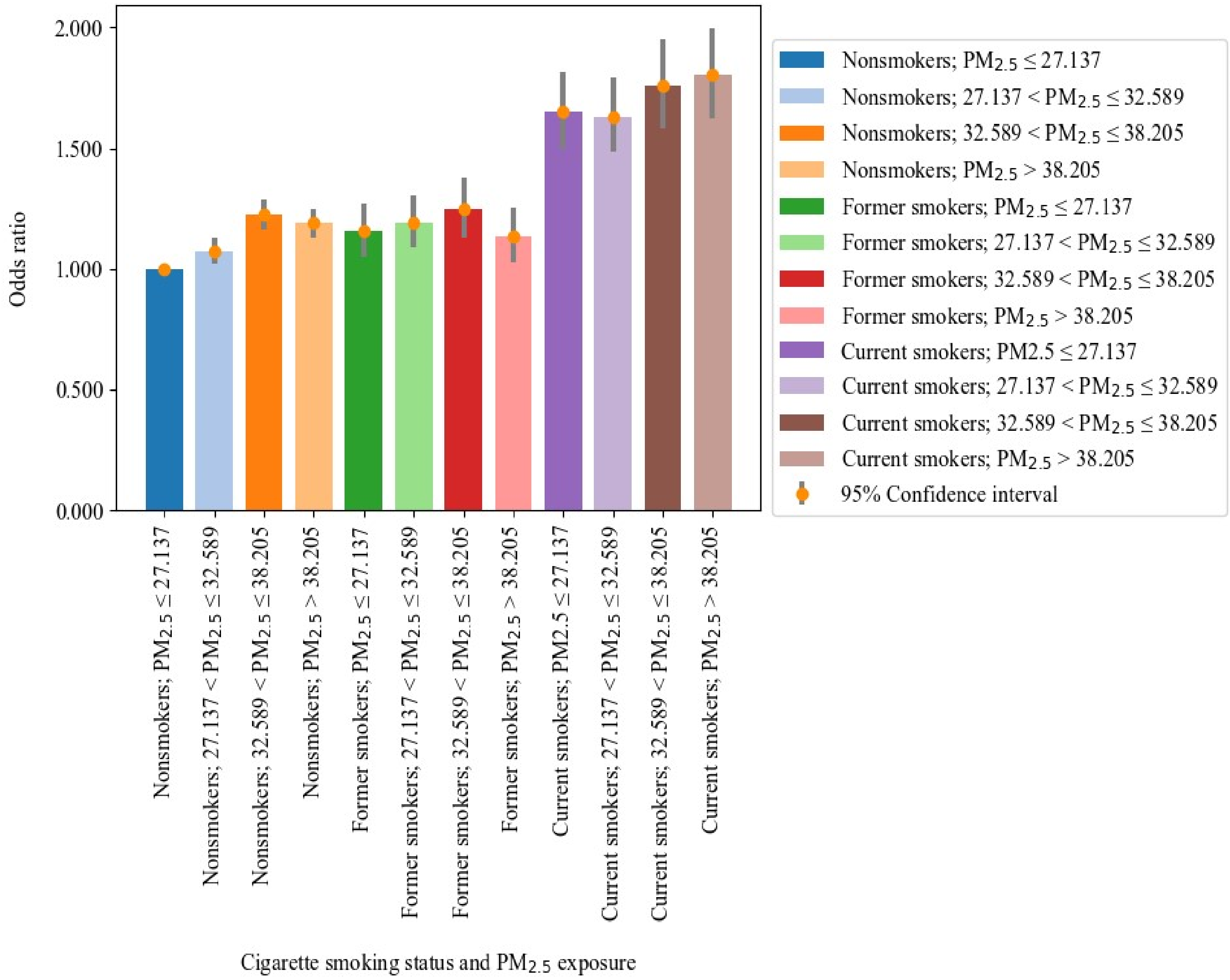

Supplementary figure 3. Risk of metabolic syndrome based on a combination of cigarette smoking and $PM_{2.5}$ exposure

Table S1. Association between the number of cigarettes smoked per week and metabolic syndrome in former and current smokers.

Variables	Former smokers			Current smokers			Current and former smokers		
	OR	95% CI	P-value	OR	95% CI	P-value	OR	95% CI	P-value
Number of cigarettes smoked per week									
<42	1								
≥42 to <70	1.129	0.904-1.411	0.2846	1.082	0.886-1.321	0.4398	1.114	0.961-1.291	0.1521
≥70 to <140	0.985	0.859-1.129	0.8288	1.142	1.003-1.301	0.0448	1.073	0.977-1.178	0.1408
≥140	1.285	1.144-1.444	<0.0001	1.603	1.415-1.817	<0.0001	1.359	1.249-1.478	<0.0001

Adjusted for $PM_{2.5}$, sex, age, BMI, alcohol intake status, exercise, marital status, educational level, secondhand smoke exposure, and duration of residence.

OR, odds ratio; CI, confidence interval; ref., reference; BMI, body mass index.
